# Capacity of proton beams in preserving normal liver tissue during proton beam therapy for hepatocellular carcinoma

**DOI:** 10.1093/jrr/rraa098

**Published:** 2020-11-03

**Authors:** Yu-Lun Tsai, Hideyuki Takei, Takashi Iizumi, Toshiyuki Okumura, Yuta Sekino, Haruko Numajiri, Hitoshi Ishikawa, Takeji Sakae, Hideyuki Sakurai

**Affiliations:** Department of Radiation Oncology, Cathay General Hospital, Taipei, Taiwan; Proton Medical Research Center, University of Tsukuba, Tsukuba, Ibaraki, Japan; Proton Medical Research Center, University of Tsukuba, Tsukuba, Ibaraki, Japan; Department of Radiation Oncology, Faculty of Medicine, University of Tsukuba, Tsukuba, Ibaraki, Japan; Proton Medical Research Center, University of Tsukuba, Tsukuba, Ibaraki, Japan; Department of Radiation Oncology, Faculty of Medicine, University of Tsukuba, Tsukuba, Ibaraki, Japan; Proton Medical Research Center, University of Tsukuba, Tsukuba, Ibaraki, Japan; Department of Radiation Oncology, Faculty of Medicine, University of Tsukuba, Tsukuba, Ibaraki, Japan; Proton Medical Research Center, University of Tsukuba, Tsukuba, Ibaraki, Japan; Department of Radiation Oncology, Faculty of Medicine, University of Tsukuba, Tsukuba, Ibaraki, Japan; Proton Medical Research Center, University of Tsukuba, Tsukuba, Ibaraki, Japan; Department of Radiation Oncology, Faculty of Medicine, University of Tsukuba, Tsukuba, Ibaraki, Japan; Proton Medical Research Center, University of Tsukuba, Tsukuba, Ibaraki, Japan; Proton Medical Research Center, University of Tsukuba, Tsukuba, Ibaraki, Japan; Department of Radiation Oncology, Faculty of Medicine, University of Tsukuba, Tsukuba, Ibaraki, Japan

**Keywords:** unirradiated liver volume, normal liver sparing, proton beam therapy, hepatocellular carcinoma

## Abstract

Unirradiated liver volume (ULV) preservation rate is an important factor associated with radiation-induced liver disease (RILD) in patients with hepatocellular carcinoma (HCC) undergoing proton beam therapy (PBT). The purpose of this study is to identify the predictors for ULV preservation and quantify the capacity of proton beams in normal liver sparing during PBT. We reviewed planning data of 92 patients with single intrahepatic HCC tumors undergoing PBT. The potential clinical and planning factors that may affect ULV preservation were involved in multiple linear regression for ULV preservation rate. The significant factors were determined to be predictors and their influences were quantified. The median ULV preservation rate was 62.08%. All the assessed clinical factors showed significant effects on ULV preservation rate: clinical target volume (CTV), *P* < 0.001; portal vein tumor thrombosis (PVTT), *P* = 0.010; left lobe tumor, *P* = 0.010. In contrast, none of the planning factors demonstrated significance. The coefficients of significant factors in multiple linear regression were 60.85 for intercept, −0.02 for CTV, −9.01 for PVTT and 8.31 for left lobe tumors. The capacity of proton beams to spare normal liver tissue during PBT for HCC is mainly affected by clinical factors. The baseline of the ULV preservation rate is 60.85%, decreasing 0.02% with each milliliter of CTV increase and 9.01% for tumors with PVTT, and increasing 8.31% for tumors limited to the left lobe. Further clinical studies should be carried out to correlate our dosimetric findings with clinical outcomes.

## INTRODUCTION

Proton beam therapy (PBT) is effective and safe in treating hepatocellular carcinoma (HCC) [1–3]. The local control rate and overall survival of the treatment are encouragingly high [4–8]. The abrupt dose falloff beyond the Bragg peak with no exit dose along the beam path confers unique dosimetric advantages on PBT for HCC [9]. It is especially feasible to apply PBT for large tumors or tumors with portal vein tumor thrombosis (PVTT) [10, 11].

Despite the advantages of PBT, radiation-induced liver disease (RILD) may still occur after treatment [6, 12–14]. Posttreatment liver decompensation is a possible cause associated with survival [15, 16]. Other than underlying liver function, radiation dose delivered to the normal liver is an important predictor of RILD [17, 18]. For PBT, the percentage volume of normal liver that is not irradiated, called unirradiated liver volume (ULV) preservation rate, independently predicts RILD in patients with HCC [17, 18]. This is the essential dosimetric parameter with respect to the area of normal liver eliminating low-dose bath that may translate into clinical outcomes [16, 17].

Understanding more about ULV preservation rates is critical. However, publications focusing on this from a dosimetric aspect are lacking. It is difficult for medical professionals to know the ideal liver doses for individual patients, and how to minimize the doses received by patients, if their fundamental knowledge is deficient. In addition, multiple factors may affect the ULV preservation rate and could be clinical or planning characteristics. The influence of each factor is different and the overall influences are complex, so clinical decisions may be more appropriate if the influence of each factor is well-understood. The aim of this study is to identify the factors significantly affecting ULV preservation, measure each of their influences on the preservation rate, and use them as predictors to quantify the capacity of proton beams in normal liver sparing during PBT to help medical professionals have an integrated concept of this issue in order to assist patients with HCC in the goal of liver preservation.

## MATERIALS AND METHODS

### Patients and clinical characteristics

With the ethical approval of our institutional review board, 92 eligible patients with HCC consecutively undergoing PBT at our institution from 2016 to 2019 were retrospectively reviewed. Each patient had a single intrahepatic HCC tumor with no prior surgery. The median clinical target volume (CTV) of the tumors was 95.36 mL. Among them, 19 patients had tumors with PVTT and 73 patients without. Tumors located in the right lobe accounted for 59.8% of all tumors. For the rest, 22.8% were in the left lobe, 13.0% across bilateral lobes and 4.4% in the caudate lobe, respectively. All patients had a median of 1032.52 mL for their normal livers. The clinical characteristics are listed in Table 1.

**Table 1:** Characteristics of clinical and planning factors

	All patients (*n* = 92) % (No.) or median (range)
**Clinical factors**	
CTV (mL)	95.36 (5.42–1634.69)
PVTT	
No	79.3% (73)
Yes	20.7% (19)
Tumor location	
Left lobe	22.8% (21)
Right lobe	59.8% (55)
Bilateral lobes	13.0% (12)
Caudate lobe	4.4% (4)
**Planning factors**	
Number of proton beams	
1	2.2% (2)
2	75.0% (69)
3	22.8% (21)
Angle between proton beams (degrees)	60.0 (0.0–180.0)

### Equipment for PBT

The machine used for treatment was a ProBEAT model (Hitachi Ltd., Tokyo, Japan) with the accelerator type of synchrotron. The treatment room included a full rotation gantry with double-scattering system and the availability of three collimators. In the beam path, a ridge filter generated spread-out Bragg peaks from 10 to 120 mm in steps of 10 mm in the depth direction, a range shifter was used to control the beam ends and a compensator was made for each beam to form a distal shape. Two dose monitors on the gantry were employed to control the absolute dose. For accessory equipment, fluoroscopy with two imaging intensifiers in the frontal and lateral views was applied for position verification. Respiratory gating using a system of laser-displacement sensors (Keyence Corp., Osaka, Japan) was employed for all patients with HCC to treat them at the end of exhalation. More details of the equipment are given in [19].

### Treatment planning and planning characteristics

The treatment planning system was the VQA (Hitachi Ltd., Tokyo, Japan). The protocol of prescribed dose involved 66 Gy(RBE) in 10 fractions, 72.6 Gy(RBE) in 22 fractions, 74 Gy(RBE) in 37 fractions and 77 Gy(RBE) in 35 fractions depending on the tumor location [19, 20]. The main scheme was to cover the entire CTV with adequate surrounding margins by the prescribed dose while sparing adjacent organs at risk (OAR) as much as possible without a fixed eligible threshold for ULV preservation rate. The margins surrounding the CTV included a general lateral margin of 9–10 mm, an additional respiratory margin of 5 mm downward and a distal margin of 5–6 mm. The treatment planning was coplanar using 1–3 ports of proton beam with 0.0–180.0 degrees between bilateral ports, where 0.0 degrees indicates only 1 port of proton beam was used. The planning characteristics are also listed in Table 1.

### ULV preservation rate

**Fig. 1.**
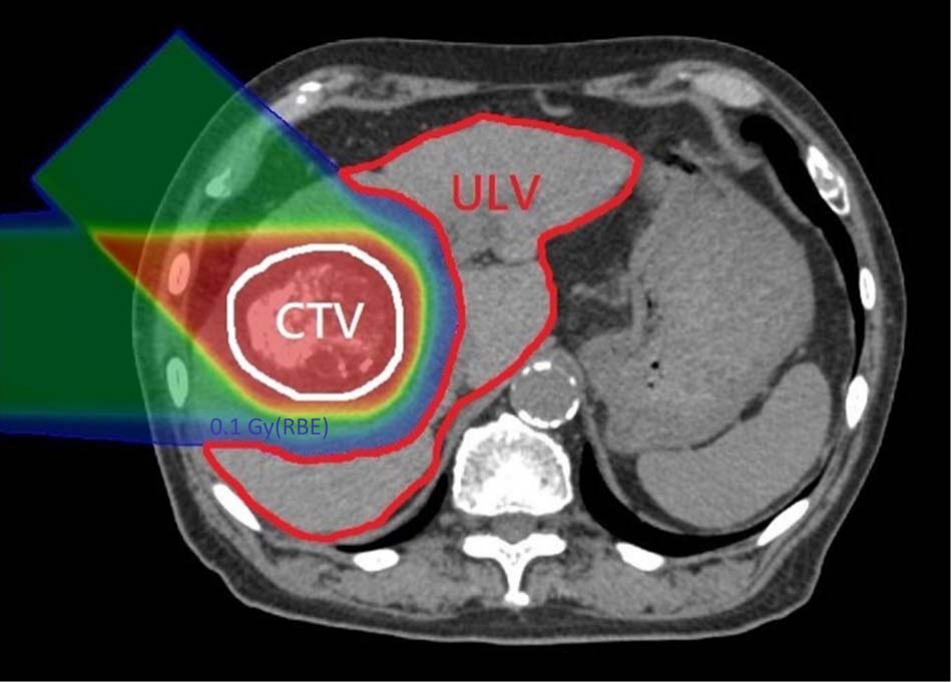
ULV preservation rate is defined as the ratio of ULV (red line) receiving a total dose <0.1 Gy(RBE) during PBT, to NLV, which is the whole liver volume minus CTV (white line).

Fig. 1 illustrates the concept of ULV and normal liver volume (NLV). ULV is defined as the liver volume that receives a total dose <0.1 Gy(RBE) during PBT and NLV is defined as the whole liver volume minus CTV. NLV is presented in the following formula:

$$ \mathrm{NLV}=\mathrm{whole}\ \mathrm{liver}\ \mathrm{volume}-\mathrm{CTV} $$

For the ULV preservation rate, the definition is the ratio of ULV to NLV, where ULV is the numerator and NLV is the denominator. It is presented in the following formula:

$$ \mathrm{ULV}\ \mathrm{preservation}\ \mathrm{rate}\ \left[\%\right]=\frac{\mathrm{ULV}}{\mathrm{NLV}}\times 100\% $$

### Data analysis

Our first goal is to identify the clinical and planning factors significantly affecting ULV preservation rate. Individual factors were statistically tested for ULV preservation rate by multiple regression analysis, and significant factors in the multiple regression analysis were determined to be the predictors for ULV preservation rate. The D_100_ and D_max_ of CTV were also involved in the statistical analysis to understand the benchmark of the results of ULV preservation rate.

Our second goal is to quantify the influences of the significant factors on ULV preservation rate using multiple regression analysis to construct a predictive model. The factors showing significance in the multiple regression analysis with their coefficients constituted the model. The final model was further verified by the model performance metrics for model evaluation and selection.

### Statistics

Multiple linear regression was used for multiple regression analysis to identify and quantify the influences of individual factors on ULV preservation rate. It was also the method for statistical analysis in which the D_100_ and D_max_ of CTV were involved. One-way analysis of variance with Bonferroni correction for multiple comparisons and independent-samples t-test were employed for subgroup analyses of tumor location. Akaike information criterion (AIC) was the model performance metric for model evaluation and selection. A *P* value < 0.05 was considered significant in the statistical tests. All of the statistical calculations and figure illustrations were applied in the R version 3.5.2., a programming language and software environment for statistical computing and graphics supported by the R Foundation for Statistical Computing.

## RESULTS

The median ULV preservation rate and ULV for all patients were 62.08% and 635.06 mL, respectively. Patients with ULV preservation rate > 40% and ULV > 350 mL accounted for the majorities, respectively with 83 and 84 patients out of 92 assessed patients.

### Factors affecting ULV preservation rate

**Fig. 2.**
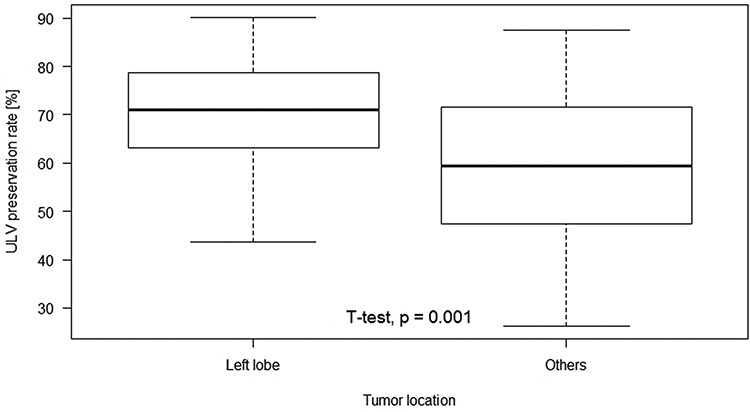
Tumors limited to the left lobe have higher ULV preservation rates.

Table 2 summarizes the discrepancies in ULV preservation rates between individuals or groups for each investigated factor. In general, only clinical factors showed statistically different rates between individuals or groups, not planning factors. For clinical factors, advanced tumors with larger CTV or PVTT resulted in worse ULV preservation rates (*P* < 0.001 and *P* = 0.010, respectively). In addition, tumor location was a significant factor, meaning that tumors located in different lobes had distinct ULV preservation rates (*P* = 0.010). Multiple comparisons indicated that the overall difference came from the pairwise differences of left and right lobes (*P* = 0.019) and of left and bilateral lobes (*P* = 0.041). Figure 2 shows a boxplot denoting the disparity of ULV preservation rates regarding tumors limited to the left and other lobes. The median preservation rate of left lobe was 70.98%, which was significantly higher than the 59.48% rate of other lobes (*P* = 0.001). For planning factors, ULV preservation rates were statistically equivalent with respect to various numbers of proton beams (*P* = 0.744) or angles between proton beams (*P* = 0.256) used in treatment planning.

**Table 2:** ULV preservation rates for different groups of clinical and planning factors

	ULV preservation rate	*P-*Value
	% Median (range)	
**Clinical factors**		
CTV (mL)		<0.001^*^
< 95.36	71.97% (43.67–90.16)	
> 95.36	51.58% (26.25–85.93)	
PVTT		0.010^*^
No	68.44% (31.73–90.16)	
Yes	48.34% (26.25–76.65)	
Tumor location		0.010^*^
Left lobe	70.98% (43.66–90.16)	
Right lobe	59.51% (26.25–87.48)	
Bilateral lobes	54.89% (26.67–80.36)	
Caudate lobe	72.30% (39.03–83.68)	
**Planning factors**		
Number of proton beams		0.744
1	56.30% (51.49–61.11)	
2	61.02% (26.25–90.16)	
3	68.60% (31.73–84.28)	
Angle between proton beams (degrees)		0.256
<60	61.11% (34.08–87.48)	
=60	58.54% (34.68–90.16)	
>60	68.28% (26.25–88.50)	

^*^Statistical significance.

### D_100_ and D_max_ of CTV

Table 3 summarizes the D_100_ and D_max_ of CTV corresponding to individual groups of factors. Basically, the results demonstrated steady CTV coverage and hotspots. The medians of D_100_ were within 90–100%, except for the plans using 1 port of beam. In addition, the medians of D_max_ were all within 100–105%. Among them, CTV was the only factor that led to a statistically significant trend of worse CTV coverage and higher hotspots when the CTV volume increased (*P* < 0.001 and *P* = 0.005, respectively). However, the absolute discrepancies were minimal. The whole picture indicates that the results of ULV preservation rates in the study were based on a benchmark of equivalent D_100_ and D_max_ of CTV.

**Table 3:** D_100_ and D_max_ of CTV for different groups of clinical and planning factors

	D_100_	*P*-Value	D_max_	*P*-Value
	% Median (range)		% Median (range)	
**Clinical factors**				
CTV (mL)		<0.001^*^		0.005^*^
< 95.36	97.52% (28.24–107.02)		102.31% (100.83–110.47)	
> 95.36	93.67% (13.91–101.67)		103.38% (99.49–120.00)	
PVTT		0.128		0.068
No	96.97% (13.91–107.02)		102.62% (100.83–114.85)	
Yes	92.41% (73.38–98.48)		104.05% (99.49–120.00)	
Tumor location		0.993		0.726
Left lobe	96.67% (67.08–101.52)		102.62% (101.36–106.22)	
Right lobe	96.14% (28.24–101.67)		102.89% (100.83–120.00)	
Bilateral lobes	93.25% (13.91–107.02)		103.24% (99.49–110.47)	
Caudate lobe	98.48% (95.69–99.31)		102.62% (100.83–106.72)	
**Planning factors**				
Number of proton beams		0.654		0.749
1	78.40% (67.27–89.53)		103.62% (103.44–103.79)	
2	97.11% (13.91–101.67)		102.89% (99.49–120.00)	
3	95.04% (58.51–107.02)		102.58% (101.67–110.47)	
Angle between proton beams (degrees)		0.041^*^		0.407
<60	95.73% (13.91–101.67)		102.89% (101.36–114.85)	
=60	97.84% (89.26–99.72)		101.65% (100.83–103.78)	
>60	96.03% (58.51–107.02)		103.03% (99.49–120.00)	

^*^Statistical significance.

### Quantification of influences on ULV preservation rate

The coefficients of significant factors in the multiple regression analysis were 60.85 for intercept (*P* < 0.001), −0.02 for CTV (*P* < 0.001), −9.01 for PVTT (*P* = 0.010) and 8.31 for left lobe tumors (*P* = 0.010). The predictive model used to quantify the influences on ULV preservation rate is then as follows:

\begin{eqnarray*}&& \mathrm{ULV}\;\mathrm{preservation}\kern0.17em \mathrm{rate}\left[\%\right]=60.85-0.02\left(\mathrm{CTV}\right)-9.01 \nonumber \\ && \left(\mathrm{PVTT}\right)+8.31\left(\mathrm{left}\kern0.17em \mathrm{lobe}\right) \nonumber\end{eqnarray*}

where PVTT and left lobe tumor are yes–no categorical predictors, and CTV is a continuously numerical predictor.

**Fig. 3.**
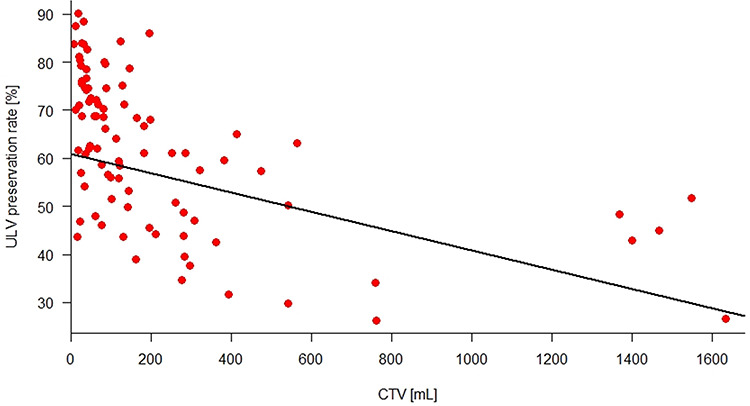
The scatter diagram to demonstrate the correlation between ULV preservation rate and CTV with regression line (straight black line). The baseline of the ULV preservation rate is 60.85%, decreasing 0.02% with each milliliter of CTV increase and adjusted by the tumor location and PVTT status.

**Fig. 4.**
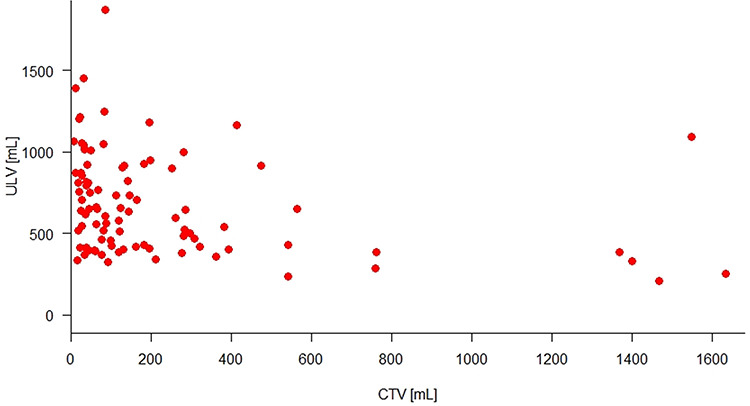
ULV tends to decrease corresponding to CTV increase.

**Fig. 5.**
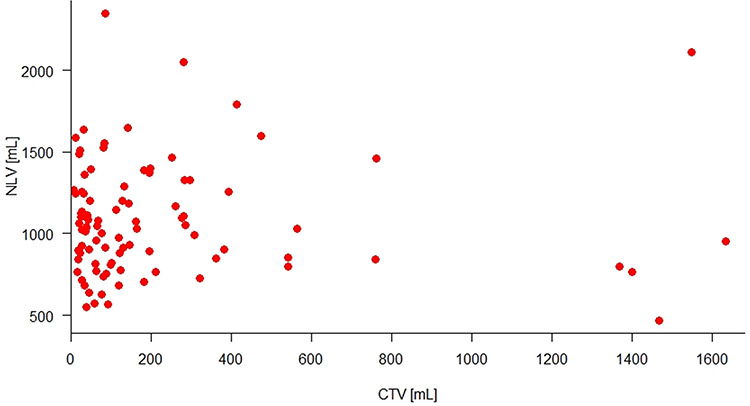
NLV has no correlation with CTV.

Fig. 3 illustrates how the ULV preservation rate decreased in accordance with the CTV increase. The baseline of ULV preservation rate, which represents the CTV as 0 mL, was 60.85% and decreased 0.02% with each CTV increase per milliliter. This precise prediction (*P* < 0.001) was probably related to the predictions of ULV by CTV (*P* = 0.012) and NLV by CTV (*P* = 0.678), respectively presented in Figs 4 and 5, as ULV preservation rate is a volumetric parameter derived from the ratio of ULV to NLV.

According to Fig. 3, the majority of patients had tumors with CTV < 800 mL, which means the tumors were smaller than ~11.5 cm in diameter if they were spherical. For this subgroup, the multiple regression analysis showed that the coefficients of significant factors were 57.91 for intercept (*P* < 0.001), −0.055 for CTV (*P* < 0.001) and 8.97 for left lobe tumors (*P* = 0.003). PVTT was no longer a predictor for ULV preservation rate (*P* = 0.489). The predictive model for CTV < 800 mL is then as follows:

\begin{eqnarray*} && \mathrm{ULV}\;\mathrm{preservation}\;{\mathrm{rate}}_{\mathrm{CTV}<800\;\mathrm{mL}}\left[\%\right]=57.91-0.055 \nonumber \\ && \qquad\left(\mathrm{CTV}\right)+8.97 \left(\mathrm{left}\kern0.17em \mathrm{lobe}\right) \nonumber\end{eqnarray*}

### Verification of influence model by AIC

For the general model, the AIC value of the original model was 730.44, which was smaller than the AIC values of the models without CTV (750.03), PVTT (736.23) or left lobe tumor (735.28). The original model with CTV, PVTT and left lobe tumor as predictors was verified. For the model of CTV < 800 mL, the AIC value was 677.85, which was smaller than the AIC values of the models with PVTT (678.43) or without CTV (718.37) or left lobe tumor (685.79) as predictors. The original model for CTV < 800 mL was also verified.

## DISCUSSION

The predictive model from our work provides an integrated estimation for quantifying the capacity of proton beams in preserving normal liver tissue during PBT for HCC. The ULV preservation is >60% in general and is affected by the CTV, PVTT and tumor location. Among them, CTV is a volumetric parameter that is the most predominant factor and the influence is in accordance with volume increase per milliliter. For the other model of CTV < 800 mL, CTV even overtakes the importance of PVTT and dominates the ULV preservation. Our discovery could help medical professionals gain more understanding of normal liver sparing and may improve clinical decisions such as the prescribed dose, efforts to minimize the doses to the liver and the determination of whether to employ PBT. This might be helpful in establishing more appropriate treatments for patients in clinical practice.

According to our findings, clinical factors have more influence on ULV preservation rate than planning factors, meaning that the amount of normal liver tissue patients can retain depends mainly on their individual disease status. However, this general result originates from collective data instead of from an individual patient. Furthermore, RILD is not the only possible complication of PBT. Although incidences are low, it is possible to develop gastrointestinal hemorrhage, bile duct stenosis, chest wall pain and rib fractures [21–24]. In clinical practice, it is still crucial to minimize the dose to the liver and other OAR individually during treatment planning.

In the history of radiotherapy for HCC, the dose–volume parameters used to predict RILD evolved for various types of radiotherapy modality. For photon beam therapy, mean liver dose and V_30Gy_ appeared to be the useful parameters in conformal radiotherapy [25, 26]. The normal tissue complication probability (NTCP) models involving mean liver dose and generalized equivalent uniform dose were also developed to estimate the risk of RILD [27, 28]. The concept of normal liver sparing in terms of achieving a certain amount of preserved liver receiving a dose under a certain threshold arose in the era of stereotactic body radiation therapy (SBRT). The thresholds of 800 mL < 18 Gy and 700 mL < 15 Gy were raised concerning liver SBRT for unresectable primary HCC and liver metastases, respectively [29, 30].

With regard to PBT, new evidence supporting the importance of ULV has been published [16, 17, 31]. A study by Sanford *et al*. compared the overall survival of patients receiving PBT vs photon beam therapy for HCC. They hypothesized that the Bragg peak phenomenon inherent to protons, which eliminates the low-dose bath distal to the target beam path associated with photons, translates into improved survival, with a median of 31 vs 14 months [16]. However, despite the authors’ conclusion that the survival benefit may be driven by a decreased incidence of liver decompensation, caution must be taken in making this causal inference before a prospective randomized clinical trial proves it [31]. Until now, the most irrefutable fact has been the correlation between liver dose and RILD. A study by Hsieh *et al*. proved the ratio of ULV to standard liver volume (ULV/SLV) derived from body surface area (a concept similar to the ULV preservation rate in our study) independently predicts RILD in patients with HCC undergoing PBT instead of mean liver dose [17]. Further prospective studies involving ULV/SLV or ULV preservation rate have potential and are encouraged.

In our study, we assessed absolute liver volumes, including ULV and NLV, as a dosimetric study so that physical parameters were better entities for analysis. In another clinical study, Hsieh *et al*. adopted SLV in RILD estimations [17]. Lee *et al*. proposed using individualized constraints with SLV for patients with NLV < 800 mL [32]. Schaub *et al*. used functional liver volume derived from ^99m^Tc-sulfur colloid-single photon emission computed tomography/computed tomography scans as complementary predictors of hepatotoxicity [33]. It is feasible to apply an additional ratio between physical and biomedical volumes to integrate the dosimetric results of our study in clinical applications.

Patients with advanced HCC have worse clinical outcomes. In spite of the effectiveness of PBT, the median survival of patients with large tumors or PVTT is between 1 and 2 years [34, 35]. In contrast, patients with tumor diameters of <10 cm that are >2 cm away from the porta hepatis or digestive tract survive a median of nearly 3 years [36, 37]. Although this is not caused by the treatment but the disease status itself, patients with large tumors or PVTT also have worse ULV preservation rates during PBT. It is difficult to preserve normal liver tissue in cases of large and deep tumors. This kind of ‘double trouble’ regarding survival and the risk of RILD requires more effort in the management of advanced tumors.

The discovery of tumor location affecting normal liver preservation is noteworthy. We find that a liver with a left lobe tumor can be preserved more successfully. The ULV preservation rate is ~70%, compared with tumors in the right or bilateral lobes with ULV preservation rates < 60%. Regarding the caudate lobe, the ULV preservation rate in our study was high, but the number of patients was only four. Conclusions of liver preservation for this specific location require more analyzed data. Nevertheless, as a discrepancy in ULV preservation rate >10% exists corresponding to tumor location, clinical outcomes concerning tumors in different lobes may be worthy of further study.

This study has some limitations. First, it was a dosimetric study using planning data to untie the complexity of ULV preservation during PBT for patients with HCC. Although the results were clear and meaningful, it was a planning study yielding logical guidance originating from numerical data. Radiotherapy is carried out for better clinical outcomes of patients rather than merely for better treatment planning parameters. Therefore, further studies correlating our results to clinical outcomes to examine whether our dosimetric inference will finally lead to clinical significance are needed. Second, planning factors did not demonstrate significance in the study. Care should be taken in interpreting this result. Being unable to demonstrate significance is not equivalent to being able to prove insignificance. Due to the retrospective nature of this study, the result does not provide evidence to totally exclude the potential importance of planning factors. Third, despite the quantification of the influences on ULV preservation rate based on steady CTV coverage and hotspots and the fact that the absolute discrepancies in D_100_ and D_max_ were minimal, the medians of D_100_ and D_max_ were not exactly the same between groups and even showed statistical significances with regard to different sizes of CTV. The larger the CTV was, the worse the CTV coverage and hotspots presented. However, this situation also indicates that if the CTV coverage and hotspots are equal, which means the tumor dose is better so that the normal liver preservation is worse, the result of the correlation between ULV preservation rate and CTV will be more significant. Fourth, the patients were treated by passive scattered proton beams instead of an active beam scanning technique. Scanning beams have advantages in the out-of-field absorbed dose in the entrance region over scattered beams [38]. In addition, different treatment modalities such as carbon-ion radiotherapy and X-ray therapy would have large differences in physical characteristics, especially for the dose distributions in low-dose ranges. As this study was conducted in a single institution with only scattered proton beams, attention must be paid to further improvements in treatment techniques using different modalities, which may provide opportunities to reverse our findings.

## CONCLUSIONS

According to our results, the capacity of proton beams in preserving normal liver tissue during PBT for HCC is mainly affected by clinical factors, as opposed to planning factors. The influences are complex and involve CTV, PVTT and tumor location. The predictive model from our work helps give more understanding of the influencing effects. The ULV preservation is >60% in general, decreasing 0.02% with each milliliter of CTV increase and 9.01% for tumors with PVTT, and increasing 8.31% for tumors limited to the left lobe. These dosimetric findings support the worth of further clinical investigations assessing clinical outcomes with individual clinical factors, which may improve treatments for patients with HCC.

## CONFLICT OF INTEREST

All authors have no conflicts of interest to disclose.

## References

[1] Hong TS, Wo JY, Yeap BY et al. Multi-institutional phase II study of high-dose Hypofractionated proton beam therapy in patients with localized, Unresectable hepatocellular carcinoma and intrahepatic cholangiocarcinoma. J Clin Oncol 2016;34:460–8. 26668346 10.1200/JCO.2015.64.2710 PMC4872014

[2] Bush DA, Kayali Z, Grove R et al. The safety and efficacy of high-dose proton beam radiotherapy for hepatocellular carcinoma: A phase 2 prospective trial. Cancer 2011;117:3053–9. 21264826 10.1002/cncr.25809

[3] Hata M, Tokuuye K, Sugahara S et al. Proton beam therapy for aged patients with hepatocellular carcinoma. Int J Radiat Oncol Biol Phys 2007;69:805–12. 17524568 10.1016/j.ijrobp.2007.04.016

[4] Apisarnthanarax S, Bowen SR, Combs SE. Proton beam therapy and carbon ion radiotherapy for hepatocellular carcinoma. Semin Radiat Oncol 2018;28:309–20. 30309641 10.1016/j.semradonc.2018.06.008

[5] Hsu CY, Wang CW, Cheng AL et al. Hypofractionated particle beam therapy for hepatocellular carcinoma-a brief review of clinical effectiveness. World J Gastrointest Oncol 2019;11:579–88. 31435460 10.4251/wjgo.v11.i8.579 PMC6700034

[6] Igaki H, Mizumoto M, Okumura T et al. A systematic review of publications on charged particle therapy for hepatocellular carcinoma. Int J Clin Oncol 2018;23:423–33. 28871342 10.1007/s10147-017-1190-2

[7] Hasan S, Abel S, Verma V et al. Proton beam therapy versus stereotactic body radiotherapy for hepatocellular carcinoma: Practice patterns, outcomes, and the effect of biologically effective dose escalation. J Gastrointest Oncol 2019;10:999–1009. 31602338 10.21037/jgo.2019.08.03 PMC6776803

[8] Yoo GS, Yu JI, Park HC. Proton therapy for hepatocellular carcinoma: Current knowledges and future perspectives. World J Gastroenterol 2018;24:3090–100. 30065555 10.3748/wjg.v24.i28.3090 PMC6064962

[9] Skinner HD, Hong TS, Krishnan S. Charged-particle therapy for hepatocellular carcinoma. Semin Radiat Oncol 2011;21:278–86. 21939857 10.1016/j.semradonc.2011.05.007 PMC3230301

[10] Toramatsu C, Katoh N, Shimizu S et al. What is the appropriate size criterion for proton radiotherapy for hepatocellular carcinoma? A dosimetric comparison of spot-scanning proton therapy versus intensity-modulated radiation therapy. Radiat Oncol 2013;8:48. 23497543 10.1186/1748-717X-8-48 PMC3606425

[11] Lee SU, Park JW, Kim TH et al. Effectiveness and safety of proton beam therapy for advanced hepatocellular carcinoma with portal vein tumor thrombosis. Strahlenther Onkol 2014;190:806–14. 24589917 10.1007/s00066-014-0604-6

[12] Bush DA, Smith JC, Slater JD et al. Randomized clinical trial comparing proton beam radiation therapy with Transarterial chemoembolization for hepatocellular carcinoma: Results of an interim analysis. Int J Radiat Oncol Biol Phys 2016;95:477–82. 27084661 10.1016/j.ijrobp.2016.02.027

[13] Komatsu S, Fukumoto T, Demizu Y et al. Clinical results and risk factors of proton and carbon ion therapy for hepatocellular carcinoma. Cancer 2011;117:4890–904. 21495022 10.1002/cncr.26134

[14] Kawashima M, Furuse J, Nishio T et al. Phase II study of radiotherapy employing proton beam for hepatocellular carcinoma. J Clin Oncol 2005;23:1839–46. 15774777 10.1200/JCO.2005.00.620

[15] Chapman TR, Bowen SR, Schaub SK et al. Toward consensus reporting of radiation-induced liver toxicity in the treatment of primary liver malignancies: Defining clinically relevant endpoints. Pract Radiat Oncol 2018;8:157–66. 29426691 10.1016/j.prro.2017.10.013

[16] Sanford NN, Pursley J, Noe B et al. Protons versus photons for Unresectable hepatocellular carcinoma: Liver decompensation and overall survival. Int J Radiat Oncol Biol Phys 2019;105:64–72. 30684667 10.1016/j.ijrobp.2019.01.076

[17] Hsieh CE, Venkatesulu BP, Lee CH et al. Predictors of radiation-induced liver disease in eastern and western patients with hepatocellular carcinoma undergoing proton beam therapy. Int J Radiat Oncol Biol Phys 2019;105:73–86. 30797890 10.1016/j.ijrobp.2019.02.032

[18] Mizumoto M, Okumura T, Hashimoto T et al. Evaluation of liver function after proton beam therapy for hepatocellular carcinoma. Int J Radiat Oncol Biol Phys 2012;82:e529–35. 22284041 10.1016/j.ijrobp.2011.05.056

[19] Mizumoto M, Oshiro Y, Okumura T et al. Proton beam therapy for hepatocellular carcinoma: A review of the University of Tsukuba experience. Int J Part Ther 2016;2:570–8. 31772968 10.14338/IJPT-15-00035.2 PMC6871645

[20] Mizumoto M, Okumura T, Hashimoto T et al. Proton beam therapy for hepatocellular carcinoma: A comparison of three treatment protocols. Int J Radiat Oncol Biol Phys 2011;81:1039–45. 20888707 10.1016/j.ijrobp.2010.07.015

[21] Nakayama H, Sugahara S, Fukuda K et al. Proton beam therapy for hepatocellular carcinoma located adjacent to the alimentary tract. Int J Radiat Oncol Biol Phys 2011;80:992–5. 21543162 10.1016/j.ijrobp.2010.03.015

[22] Chiba T, Tokuuye K, Matsuzaki Y et al. Proton beam therapy for hepatocellular carcinoma: A retrospective review of 162 patients. Clin Cancer Res 2005;11:3799–805. 15897579 10.1158/1078-0432.CCR-04-1350

[23] Yeung R, Bowen SR, Chapman TR et al. Chest wall toxicity after hypofractionated proton beam therapy for liver malignancies. Pract Radiat Oncol 2018;8:287–93. 29452863 10.1016/j.prro.2017.12.007

[24] Kanemoto A, Mizumoto M, Okumura T et al. Dose-volume histogram analysis for risk factors of radiation-induced rib fracture after hypofractionated proton beam therapy for hepatocellular carcinoma. Acta Oncol 2013;52:538–44. 22950386 10.3109/0284186X.2012.718094

[25] Dawson LA, Normolle D, Balter JM et al. Analysis of radiation-induced liver disease using the Lyman NTCP model. Int J Radiat Oncol Biol Phys 2002;53:810–21. 12095546 10.1016/s0360-3016(02)02846-8

[26] Kim TH, Kim DY, Park JW et al. Dose-volumetric parameters predicting radiation-induced hepatic toxicity in unresectable hepatocellular carcinoma patients treated with three-dimensional conformal radiotherapy. Int J Radiat Oncol Biol Phys 2007;67:225–31. 17056199 10.1016/j.ijrobp.2006.08.015

[27] Prayongrat A, Kobashi K, Ito YM et al. The normal tissue complication probability model-based approach considering uncertainties for the selective use of radiation modality in primary liver cancer patients. Radiother Oncol 2019;135:100–6. 31015154 10.1016/j.radonc.2019.03.003

[28] Kobashi K, Prayongrat A, Kimoto T et al. Assessing the uncertainty in a normal tissue complication probability difference (NTCP): Radiation-induced liver disease (RILD) in liver tumour patients treated with proton vs X-ray therapy. J Radiat Res 2018;59:i50–7. 29538699 10.1093/jrr/rry018 PMC5868200

[29] Son SH, Choi BO, Ryu MR et al. Stereotactic body radiotherapy for patients with unresectable primary hepatocellular carcinoma: Dose-volumetric parameters predicting the hepatic complication. Int J Radiat Oncol Biol Phys 2010;78:1073–80. 20207492 10.1016/j.ijrobp.2009.09.009

[30] Rusthoven KE, Kavanagh BD, Cardenes H et al. Multi-institutional phase I/II trial of stereotactic body radiation therapy for liver metastases. J Clin Oncol 2009;27:1572–8. 19255321 10.1200/JCO.2008.19.6329

[31] Wojcieszynski AP, Ben-Josef E. First do no harm; how to prevent liver decompensation after radiation therapy for hepatocellular carcinoma. Int J Radiat Oncol Biol Phys 2019;105:87–9. 31422816 10.1016/j.ijrobp.2019.06.007

[32] Lee CH, Hung SP, Hong JH et al. How small is TOO small? New liver constraint is needed- proton therapy of hepatocellular carcinoma patients with small normal liver. PLoS One 2018;13:e0203854. 30204800 10.1371/journal.pone.0203854 PMC6133378

[33] Schaub SK, Apisarnthanarax S, Price RG et al. Functional liver imaging and dosimetry to predict hepatotoxicity risk in cirrhotic patients with primary liver cancer. Int J Radiat Oncol Biol Phys 2018;102:1339–48. 30170100 10.1016/j.ijrobp.2018.08.029

[34] Sugahara S, Oshiro Y, Nakayama H et al. Proton beam therapy for large hepatocellular carcinoma. Int J Radiat Oncol Biol Phys 2010;76:460–6. 19427743 10.1016/j.ijrobp.2009.02.030

[35] Sugahara S, Nakayama H, Fukuda K et al. Proton-beam therapy for hepatocellular carcinoma associated with portal vein tumor thrombosis. Strahlenther Onkol 2009;185:782–8. 20013087 10.1007/s00066-009-2020-x

[36] Fukumitsu N, Sugahara S, Nakayama H et al. A prospective study of hypofractionated proton beam therapy for patients with hepatocellular carcinoma. Int J Radiat Oncol Biol Phys 2009;74:831–6. 19304408 10.1016/j.ijrobp.2008.10.073

[37] Chadha AS, Gunther JR, Hsieh CE et al. Proton beam therapy outcomes for localized unresectable hepatocellular carcinoma. Radiother Oncol 2019;133:54–61. 30935582 10.1016/j.radonc.2018.10.041 PMC6446916

[38] Clasie B, Wroe A, Kooy H et al. Assessment of out-of-field absorbed dose and equivalent dose in proton fields. Med Phys 2010;37:311–21. 20175494 10.1118/1.3271390 PMC2803717

